# Supplementary material for: Identification of new transmembrane proteins concentrated at the nuclear envelope using organellar proteomics of mesenchymal cells
Source: Nucleus. 2019 May 29;10(1):126–43. doi: 10.1080/19491034.2019.1618175 (PMC6550788; doi:10.1080/19491034.2019.1618175)
Supplement: Supplemental Material [file kncl-10-01-1618175-s001.zip › Supplementary information/Supp Legends and Table S4-S5_revision1.docx]

**Supplementary Figure Legends**

**Supplementary Figure S1. Western blot analysis of ectopically expressed proteins in crude nuclear and cytoplasmic fractions.** C3H cells stably transduced with lentiviruses expressing V5-tagged proteins were homogenized with a syringe-based method and separated into crude nuclear and cytoplasmic fractions. Equivalent amounts of each fraction were analyzed by Western blotting and densitometry to visualize V5-tagged proteins and calnexin. The graph depicts the V5/calnexin ratio in the cytoplasmic and nuclear fractions for each cell population, normalized to their levels in the unfractionated whole cell homogenate (WCH). Approximately half of the calnexin and >80-90% of histone H2B appeared in the pellet in all cases. V5-Sec61β (pan-ER marker) was ~2-fold more concentrated in the supernatant than the pellet. Conversely, V5-Lem2 (NE marker) was ~6-fold more concentrated in the pellet than in the supernatant. The V5-tagged versions of Itprip, Mfsd10, and Smpd4 were ~2-3-fold more concentrated in the pellet than in the supernatant, whereas Arl6ip6, Tmx4 and Vrk2 were present at similar levels in the two fractions.

**Supplementary Figure S2. Immunofluorescence localization of ectopically expressed NE and ER marker proteins in C3H cells.** C3H cells either were treated with no lentivirus, or were stably transduced at low MOI with lentiviruses expressing V5-Sec61b or V5-Lem2. Shown are representative immunofluorescence images of cells co-labeled with antibodies to the V5 epitope tag, calnexin, and lamin A. DNA staining (DAPI) and a merge of V5 and calnexin labeling is shown in the right panels. Scale bars, 10μm.

**Supplementary Figure S3. Immunofluorescence images of fields of cells with ectopically expressed targets.** C3H cells were stably transduced with the indicated constructs (or in the case of Smpd4, transiently transduced) and were examined by immunofluorescence staining to detect the V5 tag and calnexin. Shown are representative lower magnification fields. Scale bars, 20μm.

**Supplementary Figure S4. Immunofluorescence localization of Arl6ip6 and Tmx4 under different cell growth or expression conditions.** (A) C3H cells stably transduced with a lentivirus expressing Tmx4-V5 at either high or low MOI were co-labeled with antibodies to the V5 epitope tag and calnexin. Shown are fluorescence images of V5 staining only (left) or V5 staining merged with calnexin labeling (right). Scale bars, 10μm. (B) Plot showing the NE/ER fluorescence ratio of Tmx4, calculated as in Fig. 4B, in the high vs low MOI populations of Fig. S3A. (C) Western blot showing level of ectopic Tmx4 expression in high vs low MOI populations, with corresponding Ponceau staining. (D) C3H cells stably transduced with a lentivirus expressing V5-Arl6ip6 were examined after plating at low or high density as indicated. Cells were co-labeled with antibodies to the V5 epitope tag and calnexin. Shown are fluorescence images of V5 staining only (left) or V5 staining merged with calnexin labeling (right). (E) Graph showing the NE/ER fluorescence ratio of V5-Arl6ip6, calculated as in Fig. 4B, in the low vs high density populations of Fig. S3D.

**Supplementary Figure S5. Comparison of the localization of V5-Smpd4 in cells expressing low vs high amounts of ectopic protein.**

Immunofluorescence images showing C3H cells examined either shortly after transduction with a lentivirus expressing V5-Smpd4 (40 hours), or selected for stable lentiviral expression (>2 weeks). Cells were co-labeled with anti-V5 and the RL1 antibody recognizing FG repeat Nups of the NPC. Small numbers of cytoplasmic foci positive for both Smpd4 and RL1 (arrows) are seen in the 40 hour cells. The number of these cytoplasmic foci is greatly increased in in the >2 week transduced cells. Most V5-labeled cytoplasmic foci are clearly co-labeled with RL1. Scale bars, 10μm.

**Supplementary Figure S6. Accessibility of ectopically expressed V5-Lem2 to antibodies after permeabilization with low vs high digitonin.** (A) Scheme depicting epitope accessibility after treatment of cells with low vs high digitonin. (B) Immunofluorescence images of C3H cells incubated with a mixture of antibodies to V5, calnexin, lamin A and DAPI after no permeabilization, or permeabilization with low or high digitonin, or with Triton X-100 as indicated. All antibiotic-selected cells showed nuclear rim staining with anti-V5 using standard Triton X-100 permeabilization. Scale bars, 10μm.

**Supplementary Figure S7. Z-stacks of confocal images of C3H cells expressing V5-tagged Itprip or Smpd4.** The Z-position of each image is indicated. C3H nuclei are very flat in the Z dimension, and equatorial sections of nuclei often contain out-of-focus light from NE surfaces above or below the focal plane. Scale bars, 10μm.

**Supplementary Figure S8. Comparison of the localization of Itprip-V5 and various endogenous LINC complex components in C3H cells.** C3H cells stably expressing Itprip-V5 were co-stained with antibodies to the V5 tag and to nesprin-1, nesprin-2, nesprin-3 or Sun2 as indicated, and were examined by Airyscan. Shown are images of LINC complex protein staining only (middle panels) or merged images of V5 and LINC complex protein staining (left panels). The right panels are high magnification views of boxed areas from the left panels, showing the staining of LINC proteins alone, V5 alone and merged images. Scale bars, 10 um.

**Supplementary Figure S9. Targeting of ectopically expressed target proteins to the NE in U2OS cells.** Cells were stably transduced with lentiviruses expressing the constructs indicated. Shown are representative immunofluorescence images, with antibody staining and representation as in Fig. S1. Scale bars, 10μm.

**Supplementary Figure S10. Diagram depicting the predicted localization and membrane topology of the proteins analyzed in this study.** The localizations are deduced from immunofluorescence microscopy and accessibility after digitonin permeabilization of C3H cells. The membrane topologies depicted are based on annotations from UniProt, CCTOP and from posttranslational modifications reported in PhosphoSitePlus, based on the expectation that phosphorylation occurs only in the cytoplasmic compartment. The N-terminus of each protein is indicated, and predicted transmembrane segments are represented by colored cylinders.

**Supplementary Table S4.** Primers used in this study.

| Construct | Forward Primer (5’ to 3’) | Reverse Primer (5’ to 3’) |
| --- | --- | --- |
| pLV-EF1a-V5-Arl6ip6 | GGGGGTGGGCCCATGTCGTTCGTGGAGAGTTGG | TCGTCGCGGCCCATTACATGAGGCACCATGCCAC |
| pLV-EF1a-Tmx4-V5 | TCGGCCCGCCCGCCGCCATGACAGGCGGCTTCTGC | CCCCCGCCCGGCCCACCAGATCCCTTGTTGGCATTCTGAC |
| pLV-EF1a-V5-Smpd4 | GGGGGTGGGCCCATGGCGTTCCCTCACCTACA | TCGTCGCGGCCCATTATCAGAGCTGGTGCAGCTTCC |
| pLV-EF1a-V5-Mfsd10 | GGGGGTGGGCCCATGGGCTGGGCAGGG | TCGTCGCGGCCCATTACTCCTCCTTGGAAGTCTCTGC |
| pLV-EF1a-V5-Vrk2 | GGGGGTGGGCCCATGGCACCAAGAAGAAAAGAGAAATATAAGC | TCGTCGCGGCCCATTAGAGAAAATACAAAGCAAGAAATACAAATATCAGC |
| pLV-EF1a-Itprip-V5 | TCGGCCCGCCCGCCGCCATGGCCATGGGGCTCTTC | CCCCCGCCCGGCCCACCGCTTTTTGGGGTAGGCTGGT |
| pLV-EF1a-V5-Lem2 | GGGGGTGGGCCCATGGCCGGCCTGTCGGAC | TCGTCGCGGCCCATTATCGCTCTGAATCAGAGAAAGAGGAAG |
| pLV-EF1a-V5-Emerin | GGGGGTGGGCCCATGGACGACTATGCGGTTTTGTCC | TCGTCGCGGCCCATTAGGGATCCATCCAGAAGGGGTT |
| pLV-EF1a-V5-Sec61B | GGGGGTGGGCCCATGCCTGGTCCGACCCC | TCGTCGCGGCCCATTATACGAACGAGTGTACTTGCCCCAA |
| pLV-EF1a-V5-Tmx4 | GGGGGTGGGCCCATGACAGGCGGCTTCTGC | TCGTCGCGGCCCATTAAGATCCCTTGTTGGCATTCTGAC |
| pLV-EF1a-Smpd4-V5 | TCGGCCCGCCCGCCGCCATGGCGTTCCCTCACCTACA | CCCCCGCCCGGCCCACCGAGCTGGTGCAGCTTCC |
| pLV-EF1a-Mfsd10-V5 | TCGGCCCGCCCGCCGCCATGGGCTGGGCAGGG | CCCCCGCCCGGCCCACCCTCCTCCTTGGAAGTCTCTGC |
| pLV-EF1a-V5-Itprip | GGGGGTGGGCCCATGGCCATGGGGCTCTTC | TCGTCGCGGCCCATTATCAGCTTTTTGGGGTAGGCTG |

**Supplementary Table S5.** Summary of V5-tagged protein expression.

| Construct | WB, anti-V5 | IF, anti-V5 |
| --- | --- | --- |
| V5-Arl6ip6 | Predicted size | NE-enriched in denser cultures. In less dense cultures, some cells show peripheral ER localization as well. |
| V5-Itprip | No expression* | N/A |
| Itprip-V5 | Predicted size | NE-enriched |
| V5-Mfsd10 | Predicted size | NE-enriched |
| Mfsd10-V5 | Predicted size | NE-enriched + juxtanuclear Golgi-like staining |
| V5-Smpd4 | Predicted size | NE-enriched |
| Smpd4-V5 | Predicted size | NE-enriched + juxtanuclear Golgi-like staining |
| V5-Tmx4 | No expression* | N/A |
| Tmx4-V5 | Predicted size | NE-enriched in the cells with low MOI. Cells with high MOI show NE-enriched + peripheral ER localization. |
| V5-Vrk2 | Predicted size | NE-enriched + peripheral ER staining. |

*These proteins are predicted to contain an N-terminal signal sequence (SignalP-5.0), cleavage of which would result in loss of the V5-tag.
