# Supplementary material for: Identification of new transmembrane proteins concentrated at the nuclear envelope using organellar proteomics of mesenchymal cells
Source: Nucleus. 2019 May 29;10(1):126–43. doi: 10.1080/19491034.2019.1618175 (PMC6550788; doi:10.1080/19491034.2019.1618175)
Supplement: Supplemental Material [file kncl-10-01-1618175-s001.zip › Supplementary information/Supplementary Figures.pptx]

## Slide 1
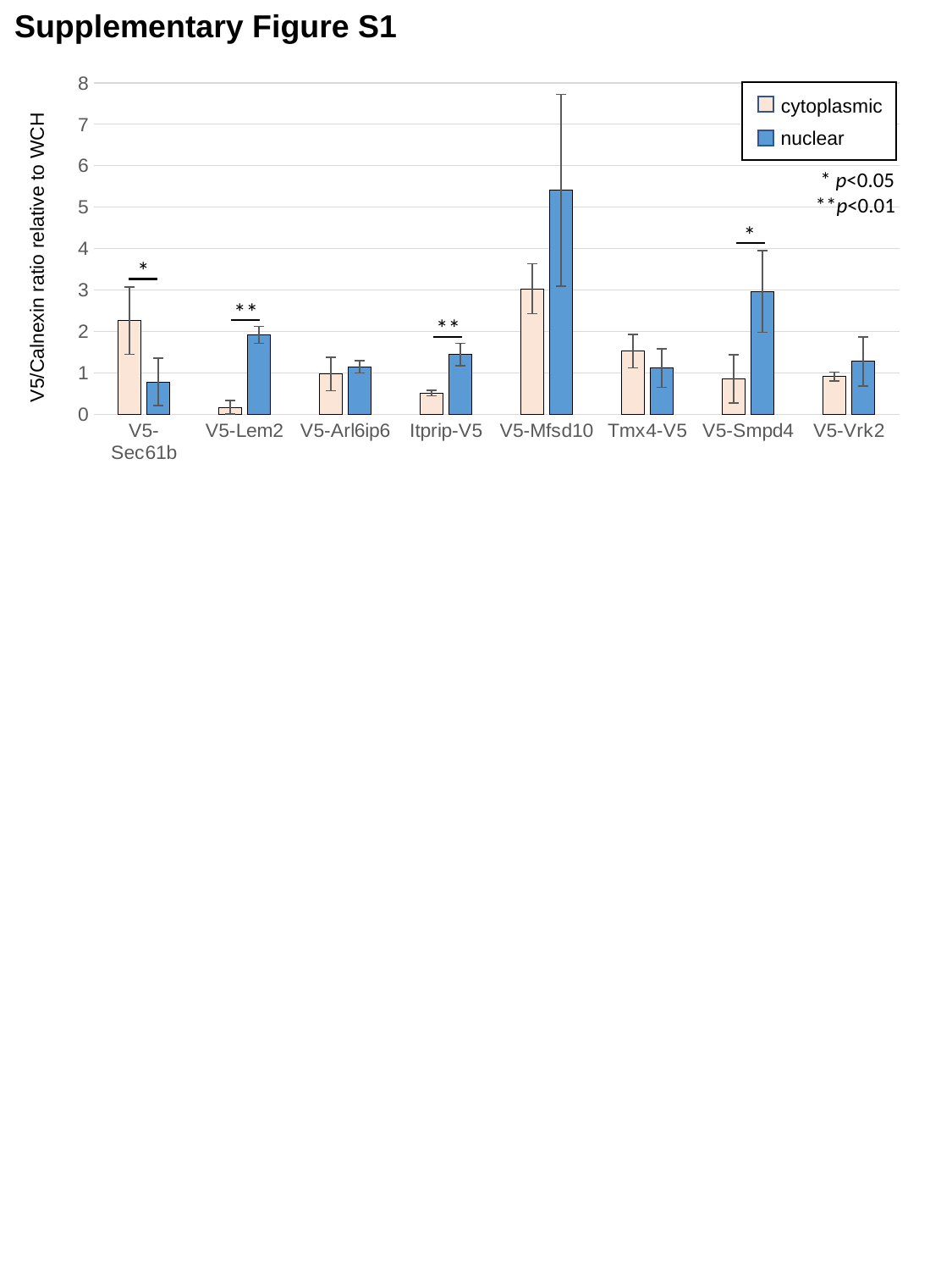

Supplementary Figure S1
### Chart
| Category | Sup | Pellet |
|---|---|---|
| V5-Sec61b | 2.257336226956644 | 0.7820252149669659 |
| V5-Lem2 | 0.17022013244517942 | 1.9180161567429652 |
| V5-Arl6ip6 | 0.9730237108553524 | 1.1447670528565363 |
| Itprip-V5 | 0.5154414096017835 | 1.4412642411554482 |
| V5-Mfsd10 | 3.028533429232556 | 5.4074907037398 |
| Tmx4-V5 | 1.5194527462956866 | 1.1146278192255004 |
| V5-Smpd4 | 0.8530731385543262 | 2.9620004675982043 |
| V5-Vrk2 | 0.9091023204471919 | 1.2746719574102774 |V5/Calnexin ratio relative to WCH
cytoplasmic
nuclear
 * p<0.05
**p<0.01
*
*
**
**

## Slide 2
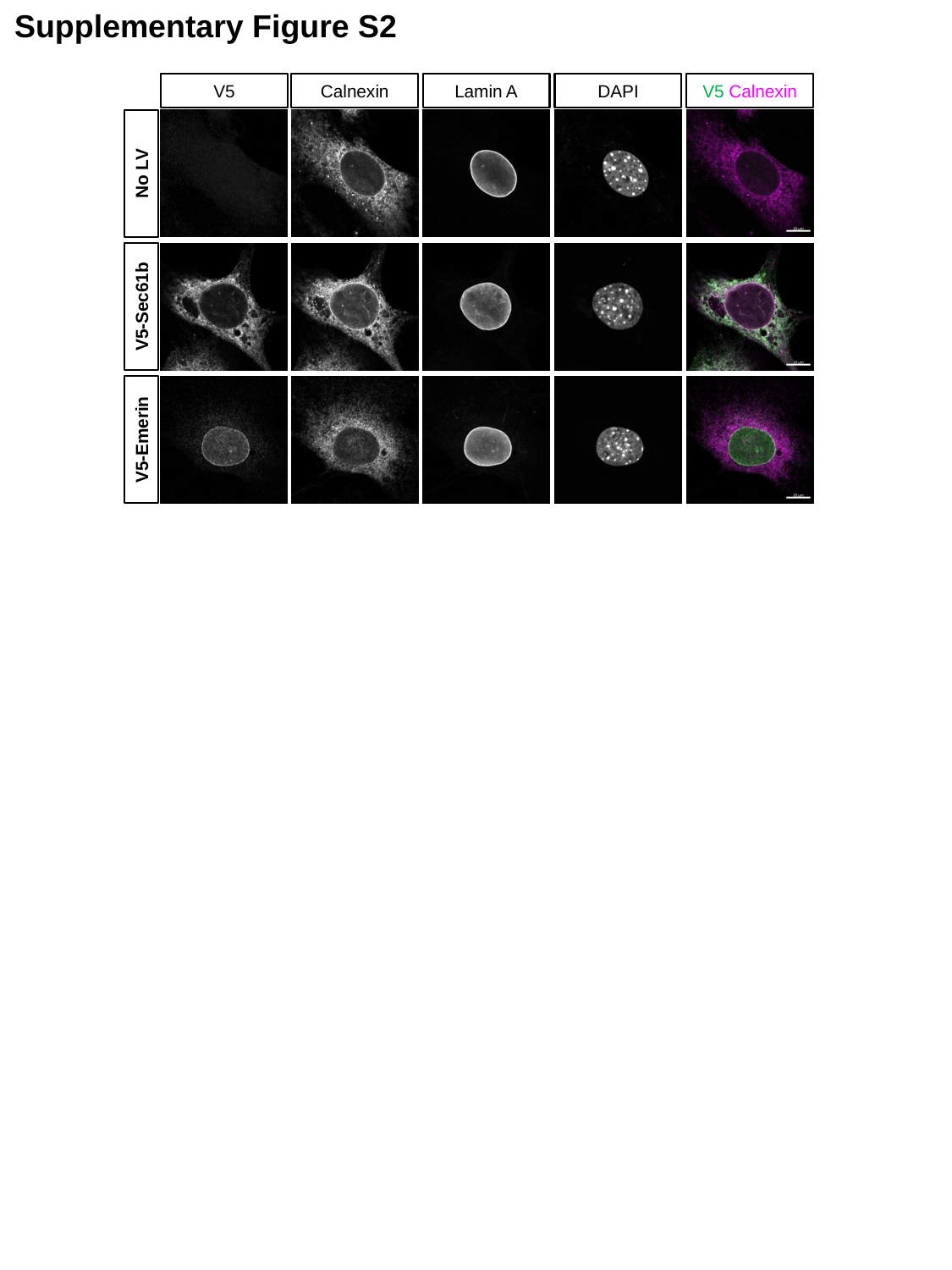

Supplementary Figure S2
V5
Calnexin
Lamin A
DAPI
V5 Calnexin
No LV
V5-Sec61b
V5-Emerin

## Slide 3
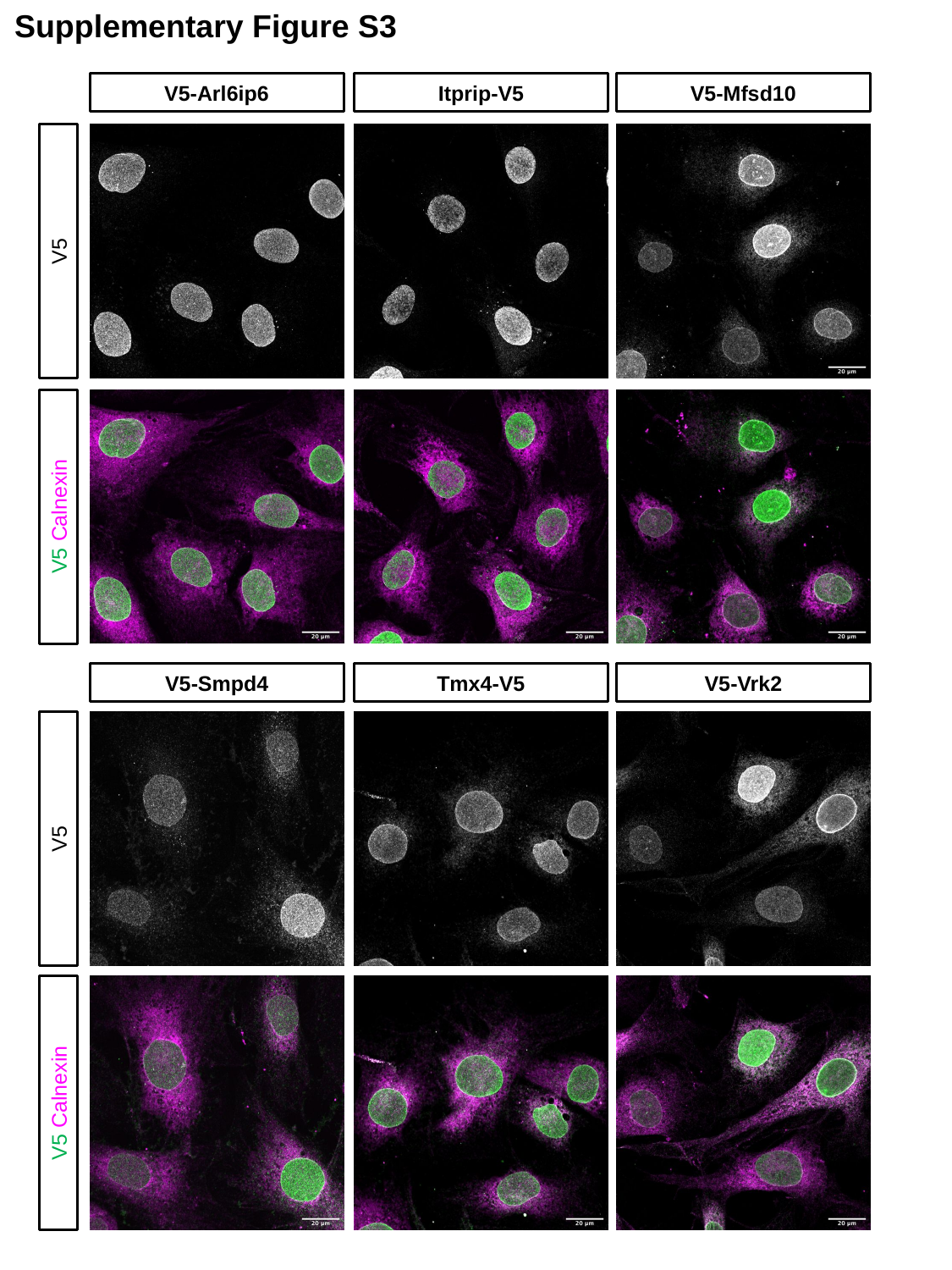

Supplementary Figure S3
V5-Arl6ip6
Itprip-V5
V5-Mfsd10
V5
V5 Calnexin
V5-Smpd4
Tmx4-V5
V5-Vrk2
V5
V5 Calnexin

## Slide 4
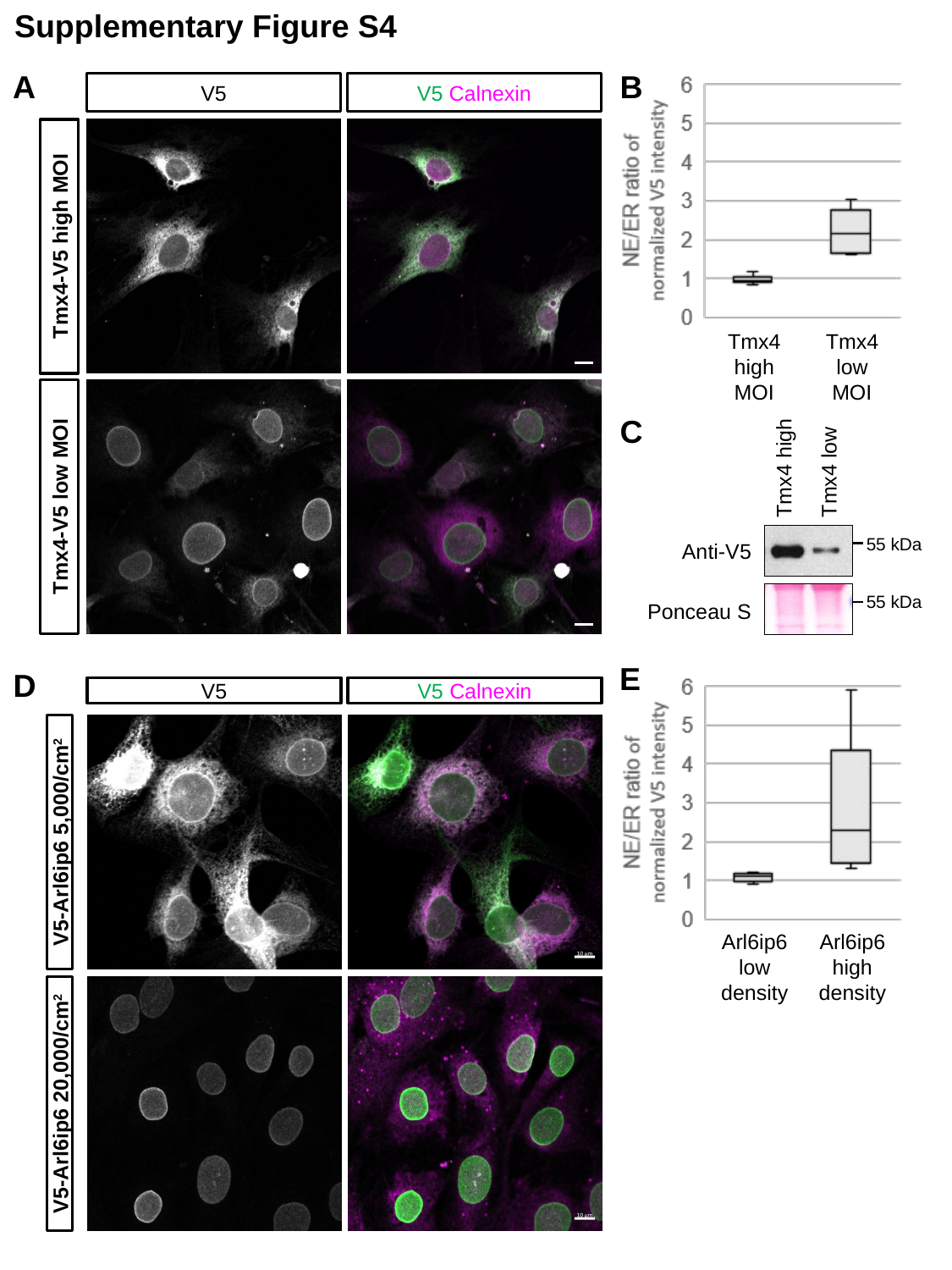

Supplementary Figure S4
A
B
V5
V5 Calnexin
Tmx4-V5 high MOI
Tmx4
high
MOI
Tmx4
low
MOI
C
Tmx4 high
Tmx4 low
Anti-V5
Ponceau S
Tmx4-V5 low MOI
55 kDa
55 kDa
E
D
V5
V5 Calnexin
V5-Arl6ip6 5,000/cm2
Arl6ip6
low
density
Arl6ip6
high
density
V5-Arl6ip6 20,000/cm2

## Slide 5
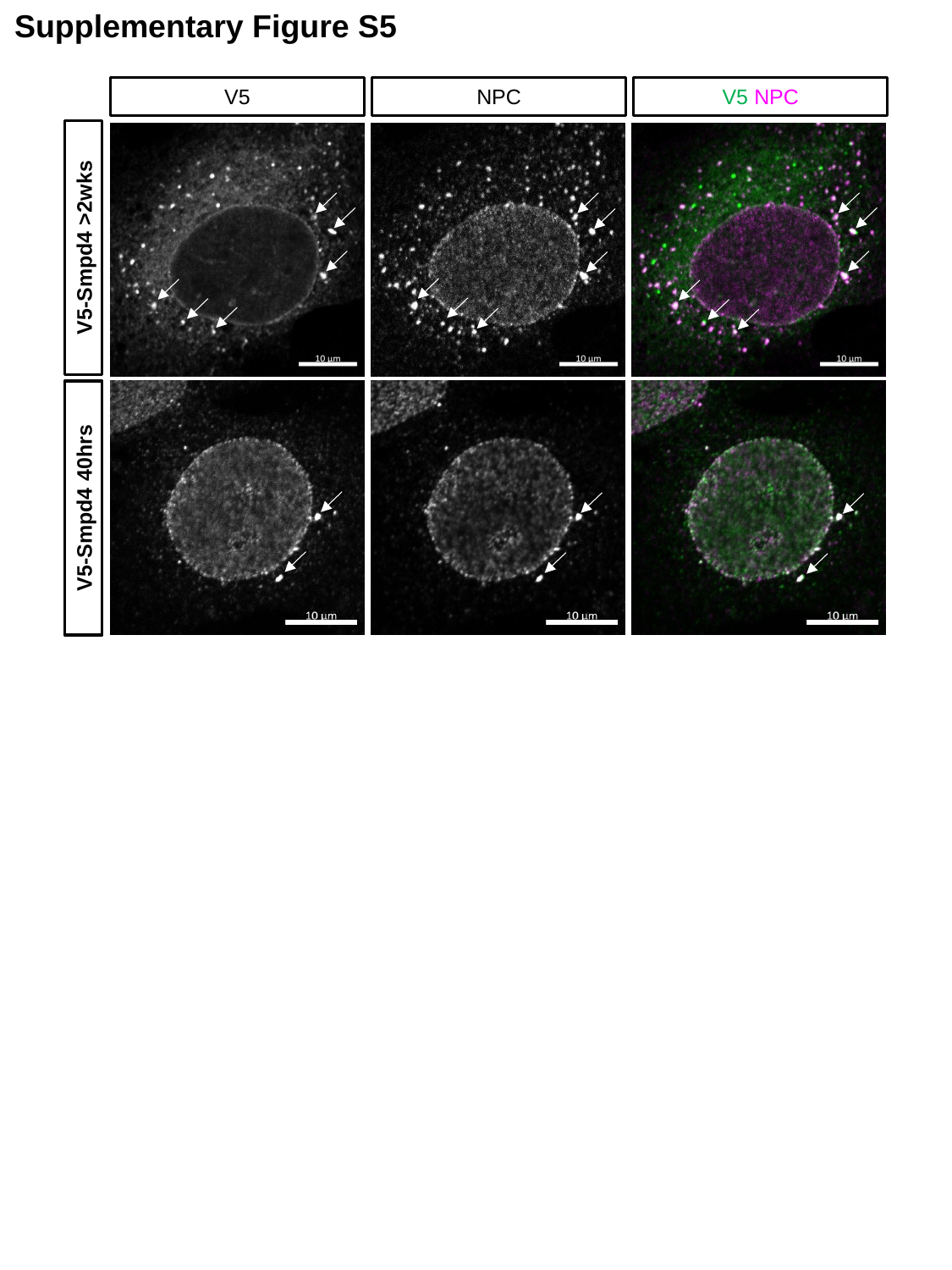

Supplementary Figure S5
V5
NPC
V5 NPC
V5-Smpd4 >2wks
V5-Smpd4 40hrs

## Slide 6
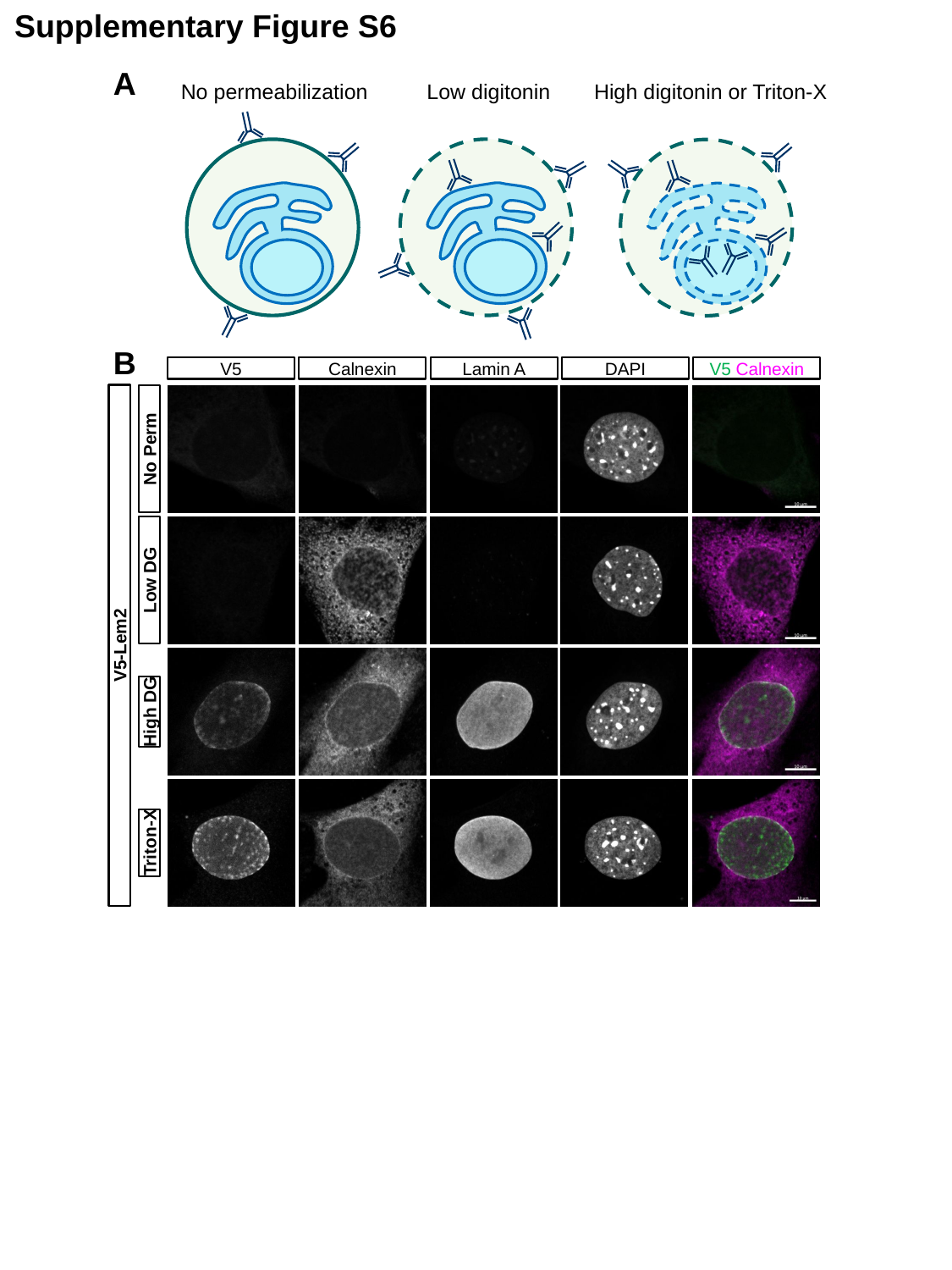

Supplementary Figure S6
A
No permeabilization
Low digitonin
High digitonin or Triton-X
B
V5
Calnexin
Lamin A
DAPI
V5 Calnexin
No Perm
Low DG
V5-Lem2
High DG
Triton-X

## Slide 7
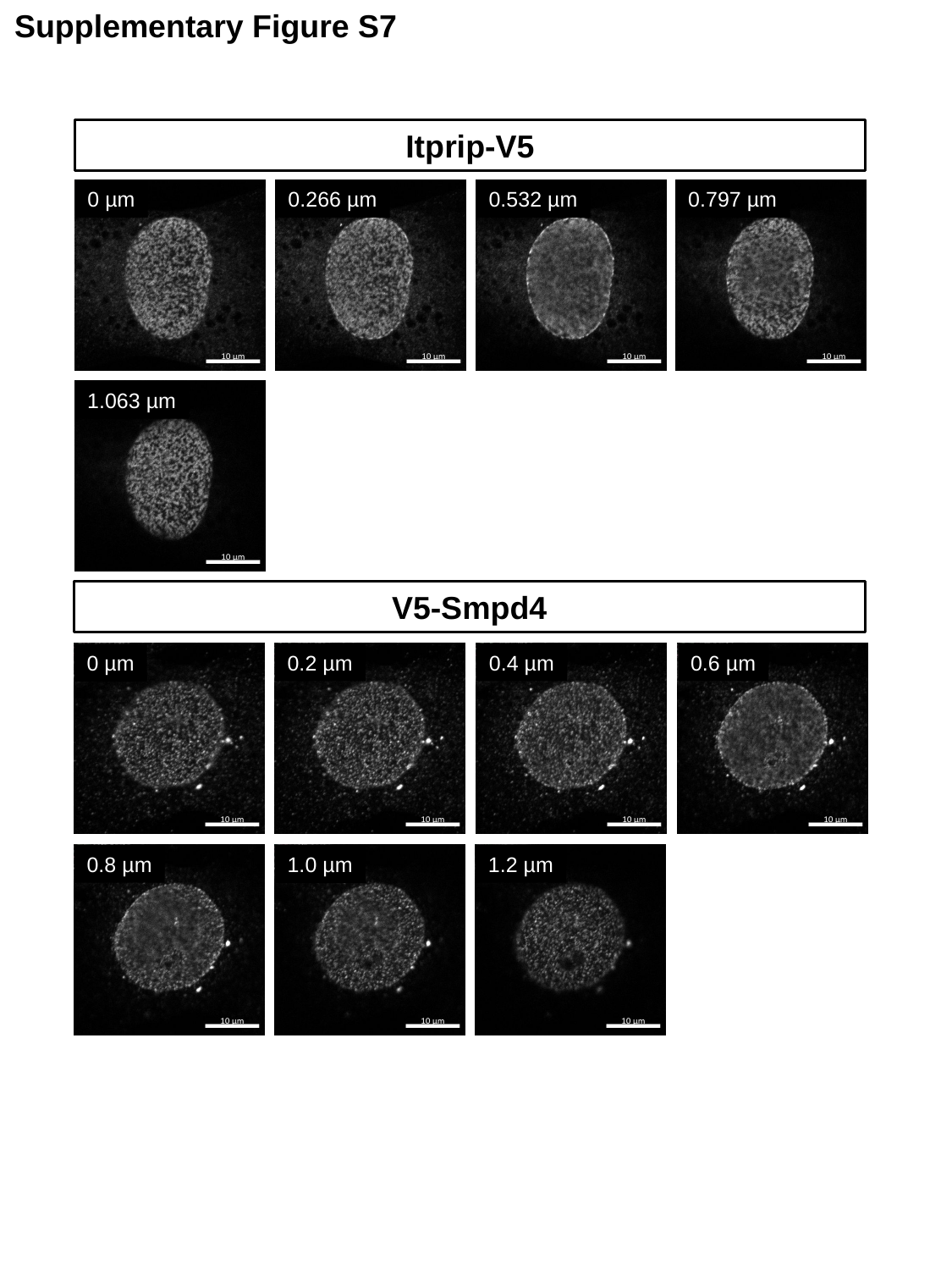

Supplementary Figure S7
Itprip-V5
0 µm
0.266 µm
0.532 µm
0.797 µm
1.063 µm
V5-Smpd4
0 µm
0.2 µm
0.4 µm
0.6 µm
0.8 µm
1.0 µm
1.2 µm

## Slide 8
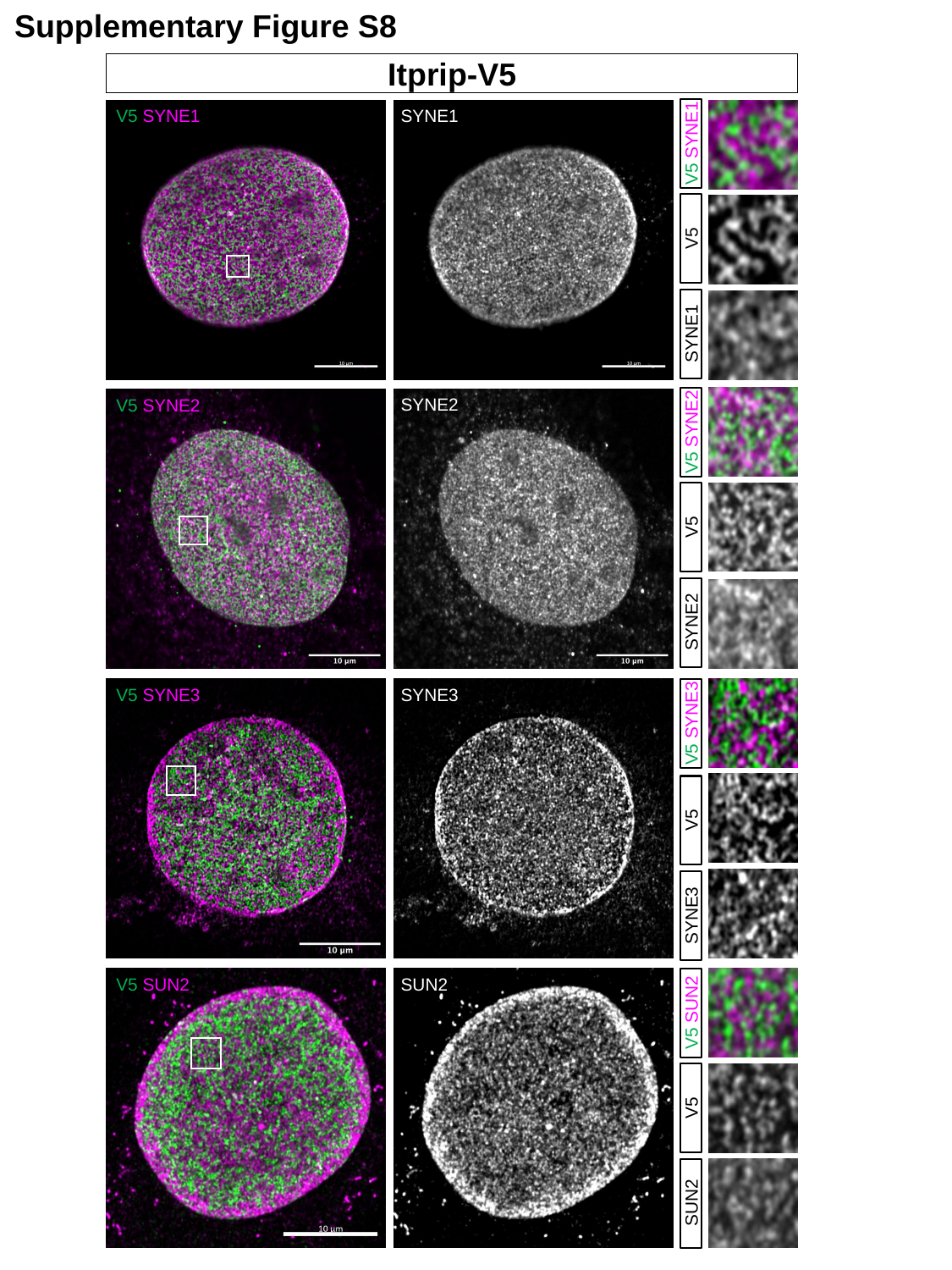

Supplementary Figure S8
Itprip-V5
SYNE1
V5 SYNE1
V5 SYNE1
V5
SYNE1
SYNE2
V5 SYNE2
V5 SYNE2
V5
SYNE2
V5 SYNE3
SYNE3
V5 SYNE3
V5
SYNE3
V5 SUN2
SUN2
V5 SUN2
V5
SUN2

## Slide 9
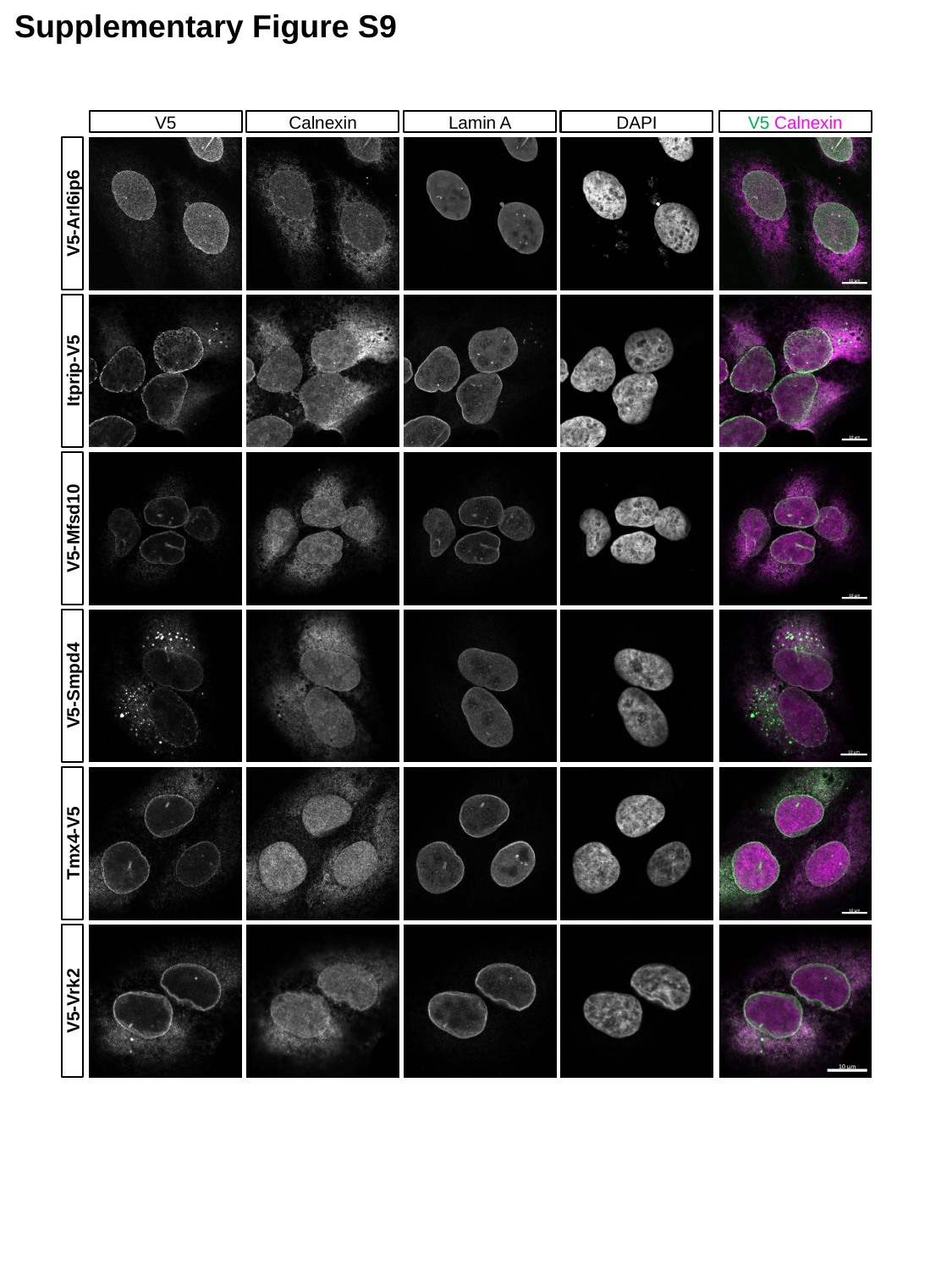

Supplementary Figure S9
V5
Calnexin
Lamin A
DAPI
V5 Calnexin
V5-Arl6ip6
Itprip-V5
V5-Mfsd10
V5-Smpd4
Tmx4-V5
V5-Vrk2

## Slide 10
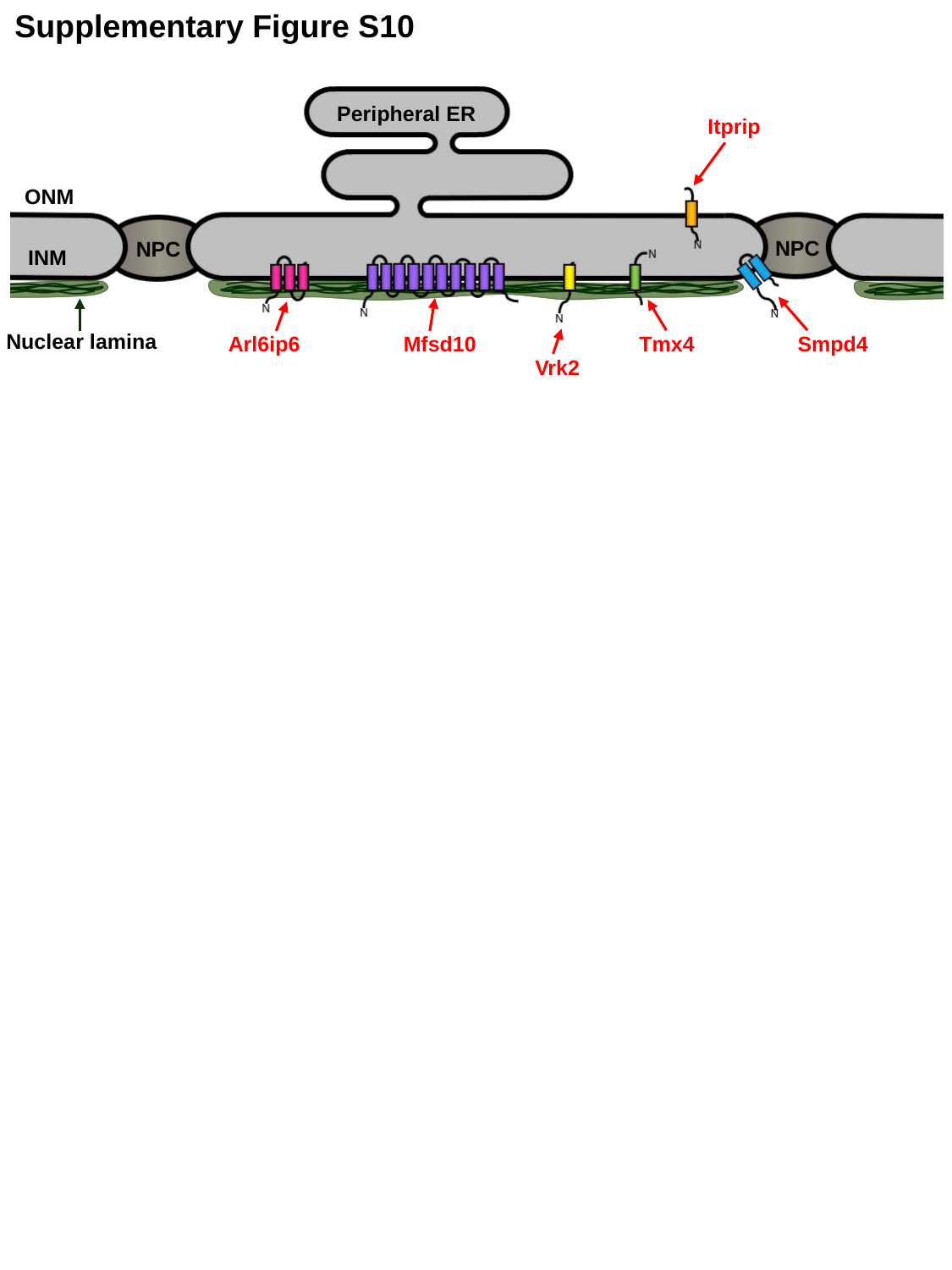

Supplementary Figure S10
Peripheral ER
Itprip
ONM
NPC
NPC
INM
Nuclear lamina
Arl6ip6
Mfsd10
Tmx4
Smpd4
Vrk2
